# Head-to-head comparison between digital and analog PET of human and phantom images when optimized for maximizing the signal-to-noise ratio from small lesions

**DOI:** 10.1186/s40658-020-0281-8

**Published:** 2020-02-21

**Authors:** Julien Salvadori, Freddy Odille, Antoine Verger, Pierre Olivier, Gilles Karcher, Pierre-Yves Marie, Laetitia Imbert

**Affiliations:** 1 Departement of Nuclear Medicine and Nancyclotep molecular imaging platform, CHRU-Nancy, Université de Lorraine, 54000 Nancy, France; 2 IADI, INSERM, UMR 1254, Université de Lorraine, 54000 Nancy, France; 3 DCAC, INSERM, UMR 1116, Université de Lorraine, 54000 Nancy, France

**Keywords:** Digital PET, Image quality, Optimization, Time-of-flight

## Abstract

**Background:**

Routine PET exams are increasingly performed with reduced injected activities, leading to the use of different image reconstruction parameters than the NEMA parameters, in order to prevent from any deleterious decrease in signal-to-noise ratio (SNR) and thus, in lesion detectability. This study aimed to provide a global head-to-head comparison between digital (Vereos, Philips®) and analog (Ingenuity TF, Philips®) PET cameras of the trade-off between SNR and contrast through a wide-ranging number of reconstruction iterations, and with a further reconstruction optimization based on the SNR of small lesions.

**Methods:**

Image quality parameters were compared between the two cameras on human and phantom images for a number of OSEM reconstruction iterations ranging from 1 to 10, the number of subsets being fixed at 10, and with the further identification of reconstruction parameters maximizing the SNR of spheres and adenopathies nearing 10 mm in diameter. These reconstructions were additionally obtained with and without time-of-flight (TOF) information (TOF and noTOF images, respectively) for further comparisons.

**Results:**

On both human and phantom TOF images, the compromise between SNR and contrast was consistently more advantageous for digital than analog PET, with the difference being particularly pronounced for the lowest numbers of iterations and the smallest spheres. SNR was maximized with 1 and 2 OSEM iterations for the TOF images from digital and analog PET, respectively, whereas 4 OSEM iterations were required for the corresponding noTOF images from both cameras. On the TOF images obtained with this SNR optimization, digital PET exhibited a 37% to 44% higher SNR as compared with analog PET, depending on sphere size. These relative differences were however much lower for the noTOF images optimized for SNR (− 4 to + 18%), as well as for images reconstructed according to NEMA standards (− 4 to + 12%).

**Conclusion:**

SNR may be dramatically higher for digital PET than for analog PET, especially when optimized for small lesions. This superiority is mostly attributable to enhanced TOF resolution and is significantly underestimated in NEMA-based analyses.

## Introduction

Positron emission tomography (PET) diagnostic procedures are commonly prescribed for a growing number of indications and are moreover increasingly repeated in the same patients for purposes of serial treatment monitoring, thereby explaining a current trend toward reducing the amount of injected activities. These changes have been facilitated not only by the concomitant advances achieved in PET camera technology [1, 2] but also by the preferential use of iterative reconstruction parameters preventing against excess noise and against a deleterious decrease in signal-to-noise ratio (SNR) and thus in the detectability of small lesions [3, 4]. These parameters are typically different from those used for the NEMA-based assessment of PET cameras, the latter favoring image contrast at the expense of noise.

The enhancement in the time-of-flight (TOF) information provided by digital PET could be particularly advantageous in this setting. The introduction of TOF imaging has indeed improved PET investigations in the last decade, with a significant enhancement in the trade-off between noise and contrast and ultimately, in small lesion detectability [5–7]. The Vereos PET/CT system (Philips, Cleveland, Ohio) was recently commercialized with a digital silicon photomultiplier (SiPM), a 1:1 coupling with small lutetium-yttrium oxyorthosilicate (LYSO) crystals together with a dramatic enhancement in TOF resolution (~ 310 ps) comparatively with analog PET systems (> 500 ps) [8]. These properties have been shown to provide excellent image quality performance according to NEMA standards [9–11] while previous clinical studies have shown that digital PET improves not only image quality but also diagnostic confidence and accuracy in oncologic diseases [12–17]. However, it is not known to what extent the properties of digital PET cameras are advantageous under routine conditions favoring SNR for image reconstruction, especially with regard to analog PET cameras equipped with comparable reconstruction algorithms and similarly optimized for SNR.

This study aimed to provide a global head-to-head comparison between digital (Vereos, Philips®) and analog (Ingenuity TF, Philips®) PET cameras equipped with equivalent reconstruction algorithms, of the trade-off between SNR and contrast through the testing of a wide-ranging number of reconstruction iterations, and with a further reconstruction optimization based on the SNR of small lesions.

## Materials and methods

### PET-CT systems

The Ingenuity TF analog PET camera (Philips, Cleveland, Ohio) operates with LYSO crystals each measuring 4 × 4 × 22 mm^3^, an axial field of view of 18 cm and 420 photomultiplier tubes [18]. The Vereos digital PET camera involves thinner LYSO crystals (4 × 4 × 19 mm^3^), a smaller axial field of view of 16.4 cm, and each of the 23,040 LYSO crystals is coupled to a SiPM containing an array of 3200 single-photon avalanche diodes (SPADs) called microcells [10, 19].

For both cameras, images were recorded in 3D mode with a 576 mm × 576 mm field of view and reconstructed with 2 mm voxels using an ordered subset expectation maximization (OSEM) algorithm [20] associated with (i) attenuation corrections provided by conventional CT-based methods, (ii) a scatter correction based on a Monte Carlo simulation [21], (iii) a random subtraction with smoothed delayed coincidence sinogram [22], (iv) a component-based normalization of the non-uniform response of PET detectors [23], and (v) a regularized version [24] of the Richardson-Lucy algorithm [25, 26] for resolution recovery with 1 iteration and 6-mm regularization kernel, with these parameters maintained unchanged for all experiments. No image filtering was currently applied and the relaxation parameter was set to 1.0, the latter controlling the magnitude of the image change induced by each iteration.

### Recording and reconstruction of PET images

Image quality parameters were averaged from three replicate 3-min recordings of phantoms placed at the center of both cameras. According to the NEMA NU2-2018 standard [27], the International Electrotechnical Commission (IEC) torso phantom was first filled with ^18^F activity concentrations set at 5.3 kBq/mL for background activity and fourfold higher in 6 spheres of 10, 13, 17, 22, 28, and 37 mm diameters. Additional images were secondly obtained from the IEC phantom with the two largest spheres filled with only water (cold spheres of the NEMA NU2-2012 standard [28]) and thirdly, from a Jaszczak phantom equipped with smaller spheres of 4, 5, 6, 8, 10, and 13 mm diameters, also with an activity set at 5.3 kBq/mL for background and fourfold higher within the spheres.

Image quality parameters were additionally compared between human digital and analog PET images recorded at 90 s per bed position, 1 h after the intravenous injection of 3 MBq kg^−1^ of ^18^F-FDG in two retrospectively selected male patients. Both patients were of comparable age (64 and 61 years) and body mass index (25 and 23 kg m^−2^), and on the digital and analog CT images, both had areas of FDG uptake within axillary adenopathies with respectively 8.2 mm and 8.8 mm diameters.

Both human and phantom images from each of the two cameras were reconstructed with and without the TOF information (TOF and noTOF images, respectively) and according to the number of OSEM iterations, ranging from 1 to 10, with the number of subsets fixed at 10.

Images were additionally reconstructed according to the reconstruction parameters proposed by the manufacturer for the NEMA image quality standards, favoring contrast with a high convergence of the reconstruction OSEM process—i.e., with 3 iterations and 17 subsets for the Vereos camera [10] and 3 iterations and 33 subsets for the Ingenuity camera [18]. When using these NEMA settings, the relaxation parameter was set at 0.5 for the Ingenuity camera and 1.0 for the Vereos camera while all remaining parameters were identical to those tested in the present study (field of view, voxel size, resolution recovery algorithm).

### Image quality metrics

Mean voxel activities were determined within spherical volumes-of-interest (VOIs) matching each phantom sphere size, with *S*_*i*_ representing the mean activity within a sphere of diameter *i*.

Sixty regions-of-interest (ROIs) were placed in the background, for each sphere size, as defined by the NEMA standard [27, 28]. The average (*B*_*i*_) and the standard deviation (*σ*_*i*_) of all voxels within the 60 background ROIs of *i* diameter were then computed.

Signal-to-noise-ratio (SNR) was computed for each phantom hot and cold sphere of *i* diameter using the following formulas [3]:

1 $$ {\mathrm{SNR}}_{\mathrm{Hot},i}=\frac{\left({S}_i-{B}_i\right)}{\sigma_i} $$

2 $$ {\mathrm{SNR}}_{\mathrm{Cold},i}=\frac{\left({B}_i-{S}_i\right)}{\sigma_i} $$

In patients, this ratio was computed with the same formula as Eq. 1, with *S* representing the mean voxel activities within a 5-mm diameter VOI centered in an adenopathy of 8 to 9 mm diameter, *B* representing the background activity obtained within a 2-cm^3^ spherical VOI placed in close proximity to the corresponding adenopathies, and *σ* is the noise computed as the standard deviation of voxel activities in the background VOI.

The contrast recovery coefficient (CRC) between each phantom element (hot and cold spheres) and its corresponding background area was computed with the following formulas [27, 28]:

3 $$ {\mathrm{CRC}}_{\mathrm{Hot},i}\left(\%\right)=\frac{\frac{S_i-{B}_i}{B_i}}{\frac{a_H}{a_c}-1}\times 100 $$

4 $$ {\mathrm{CRC}}_{\mathrm{Cold},i}\left(\%\right)=\frac{\left({B}_i-{S}_i\right)}{B_i}\times 100 $$

with $\frac{a_H}{a_c}$ representing the actual ratio of activity concentrations between spheres and background, which was set at 4 in the present instance. For the patients’ adenopathies, the actual ratio of concentrations was not known and thus only the contrast was calculated as the numerator of Eq. 3.

Relative noise (RN) was assessed through the coefficient of variation of activities from voxels setting within the 60 background ROIs of 37 mm diameter, and with the resulting values being expressed in percentages [5]:

5 $$ \mathrm{RN}\ \left(\%\right)=\frac{\sigma_{37\mathrm{mm}}}{B_{37\mathrm{mm}}}\times 100 $$

In patients, RN was estimated as the 2-cm^3^ background VOI of human images, over the mean uptake of the same areas.

The residual error for the corrections of scatter and attenuation was assessed according to the NEMA standard [27, 28] through the mean voxel activity of a cylindrical 30-mm diameter VOI centered on the lung insert of the IEC phantom (*B*_insert_) and expressed relative to mean activity of the 60 background ROIs of 37 mm diameter and thus, with the following formula:

6 $$ {\varDelta}_{error}\left(\%\right)=\frac{B_{\mathrm{insert}}}{B_{37\mathrm{mm}}}\times 100 $$

## Results

### Global analyses of the trade-off between SNR and CRC

**Fig. 1:**
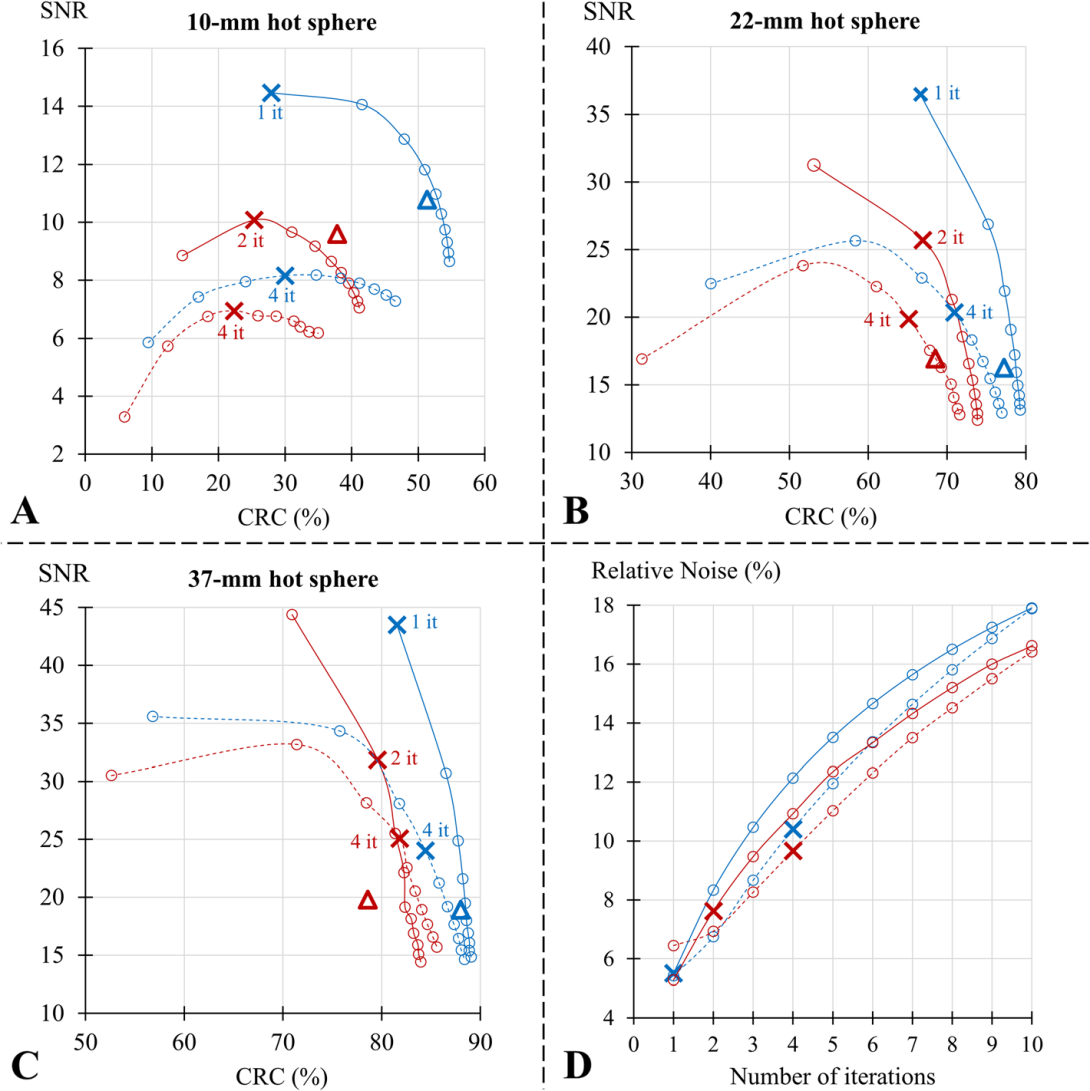
Results from the IEC phantom with the comparisons between digital PET (blue lines) and analog PET (red lines) and for images reconstructed with the TOF information (TOF, solid lines) and without the latter (noTOF, dashed lines) of (1) the relationships between signal-to-noise-ratio (SNR) and contrast recovery coefficients (CRC), according to the number of OSEM iterations involved in the image reconstruction process and for the hot spheres of 10 mm (**a**), 22 mm (**b**), and 37 mm (**c**) diameters and (2) the relative noise as a function of the number of OSEM iterations (**d**). Points corresponding to the optimal number of iterations (i.e., those maximizing SNR of the 10-mm diameter sphere) are depicted by crosses (4 iterations (it) for the noTOF images from both cameras, and 1 and 2 iterations for the TOF images from Vereos and Ingenuity, respectively), and results from NEMA-based reconstruction are represented with blue triangles for the Vereos and red triangles for the Ingenuity images

This trade-off was analyzed for three representative hot spheres (10-mm, 22-mm, and 37-mm) of the IEC phantom, through curves representing the evolution of SNR according to CRC as a function of the number of iterations, and in a comparative manner between TOF and noTOF images and between the 2 cameras (Fig. 1a-c). SNR and CRC values are also given in Supplemental Tables 1 and 2, respectively, for all spheres visible with both cameras (i.e., with a ≥ 8-mm diameter).

**Fig. 2:**
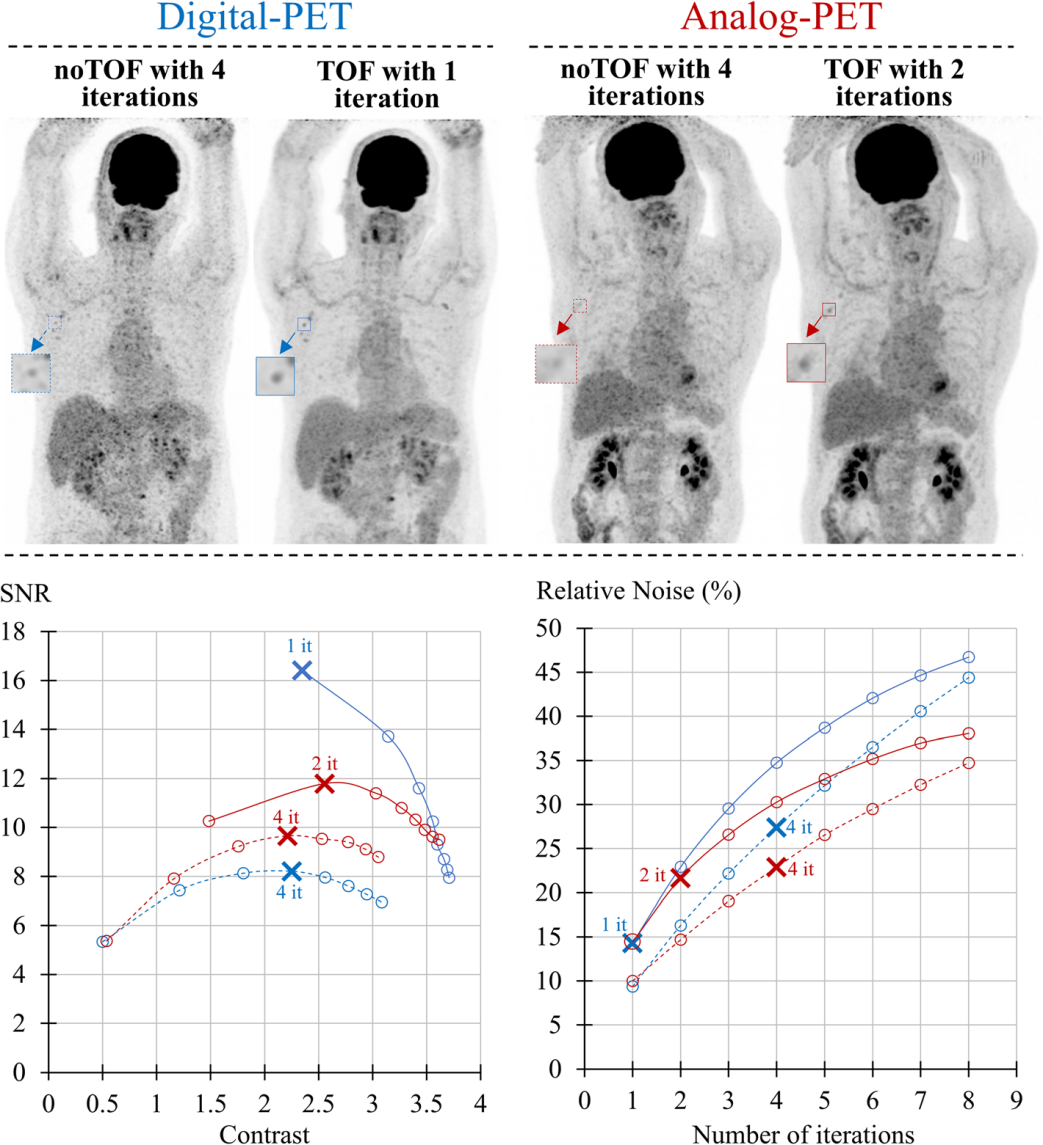
Results from human images with (1) in the upper panel, maximal intensity projections for human PET images reconstructed with the parameters providing the maximal signal-to-noise ratio (SNR) for small elements (4 iterations for noTOF images and 1 and 2 iterations for TOF images for digital and analog PET, respectively); (2) in the lower panels, the comparisons between digital PET (blue lines) and analog PET (red lines) and for images reconstructed with the TOF information (TOF, solid lines) and without the latter (noTOF, dashed lines), of the relationships between SNR and contrast recovery coefficients (CRC) for the adenopathies of 8 to 9 mm in diameter (lower left panel) and of relative noise according to the number of iterations involved in the image reconstruction process (lower right panel). Crosses are defined in the legend of Fig. 1

For all CRC values, the corresponding SNR levels were significantly higher when comparing TOF with noTOF images from the same camera, as well as when comparing the TOF images from the Vereos digital camera with those from the Ingenuity analog camera. These differences were particularly pronounced for the smallest hot spheres, in particular the 10-mm sphere, and when using a low number of iterations and thus, in conditions favoring SNR (left portions of the curves). In contrast, these differences were much less significant when using a high number of iterations and thus, in conditions favoring CRC (right portions of the curves). Note that in these latter conditions, the TOF-SNR and TOF-CRC values converged to the noTOF values. In addition, as evidenced in Fig. 2, the SNR/CRC curves obtained with the 8- to 9-mm adenopathies from the human PET exams were very similar to those documented with the 10-mm sphere of the IEC phantom (Fig. 1a).

Finally, as evidenced in Figs. 1d and 2, and for all OSEM iterations, the relative noise was higher when comparing TOF with noTOF images from the same camera, as well as when comparing the TOF images obtained with the Vereos camera with those from the Ingenuity camera.

### Image reconstruction optimized for the SNR of small elements and comparison with NEMA-based reconstructions

As evidenced in Figs. 1a and 2, the SNR of small elements (10-mm spheres and adenopathies of 8- to 9-mm diameter) was maximized with 1 and 2 OSEM iterations for digital and analog PET respectively, whereas 4 OSEM iterations were required for the corresponding noTOF images from both cameras.

On the phantom TOF images optimized for SNR (i.e., those reconstructed with 1 iteration for digital PET and 2 for analog PET), digital PET exhibited an almost 30% lower noise level (Fig. 1d) than that of analog PET, as well as a slightly higher CRC with relative increases ranging from 0-2% for the 4 largest hot spheres to 10% for the smallest 10 mm sphere (see the corresponding points on the curves from Fig. 1a-c). Consequently, from the largest to the smallest spheres, the TOF images from digital PET provided a 37% to 44% higher SNR than those of analog PET (Fig. 1a-c).

The relative differences in SNR between the 2 cameras were conversely much lower for the noTOF images optimized for SNR (from − 4 to + 18%, see the corresponding points on the curves from Fig. 1). For these noTOF images, however, digital PET exhibited higher values for both noise (+ 8%, Fig. 1d) and contrast (+ 34%, + 9%, and + 3% for the 10-mm, 22-mm, and 37-mm spheres respectively, see Fig. 1a-c), presumptively in keeping with a lower count sensitivity and a better spatial resolution, respectively (see the “Discussion” section).

In addition, in TOF images reconstructed according to NEMA standards, the relative differences in SNR between the 2 cameras were rather low, ranging from −4 to + 12% according to sphere size (see the corresponding levels depicted by triangle symbols in Fig. 1 and values given in Supplemental Table 1). In this latter instance, however, the limited advantage regarding SNR was counterbalanced by a marked advantage for CRC as compared with analog PET (i.e., with the increase in relative differences ranging from + 12% for the 37-mm sphere to + 36% for the 10-mm sphere, see Fig. 1a-c and Supplemental Table 2).

### Cold contrast and relative error

**Fig. 3:**
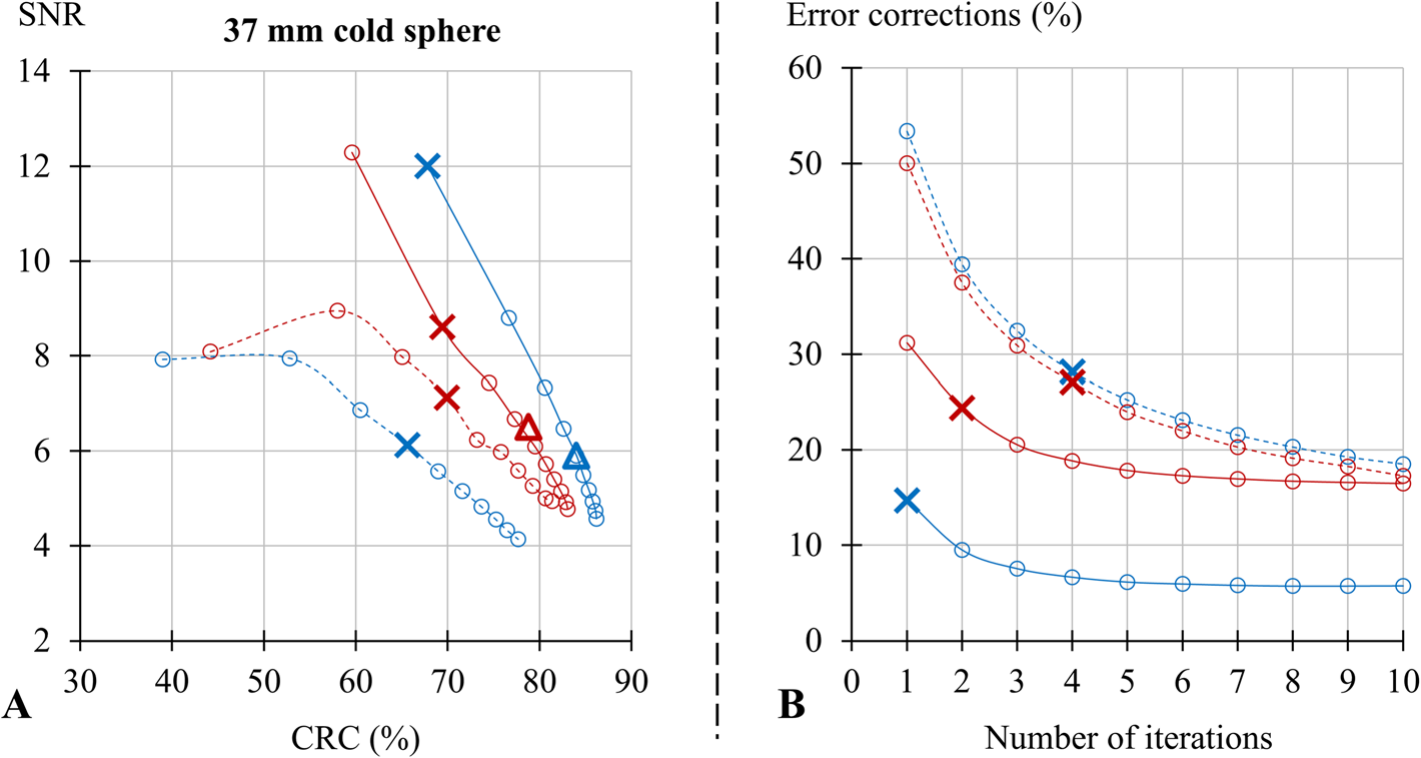
Results from the IEC phantom and lung insert with the comparisons between digital PET (blue lines) and analog PET (red lines) and for images reconstructed with the TOF information (TOF, solid lines) and without the latter (noTOF, dashed lines) of (1) the relationships between signal-to-noise-ratio (SNR) and contrast recovery coefficients (CRC) for the 37-mm cold sphere (**a**), and (2) the % error correction as a function of the number of OSEM iterations (**b**). Crosses and triangles are defined in the legend of Fig. 1

As evidenced in Fig. 3a, the SNR-CRC trade-off documented with the large cold sphere was better with analog PET than with digital PET for the noTOF images and, at the opposite, better with digital PET for the TOF images. On noTOF images optimized for SNR (4 iterations for both cameras), digital PET exhibited a 14% lower SNR than analog PET. However, on the TOF images optimized for SNR (1 iteration for digital PET and 2 for analog PET), the SNR increased by 97% for the digital PET and by only 21% for the analog PET, leading to a 40% higher level of SNR for digital PET than for analog PET.

As shown in Fig. 3b, the residual error in the corrections applied for scatter and attenuation was very similar between the noTOF images from both cameras. This residual error was however markedly decreased on the corresponding TOF images, especially for digital PET, the latter showing a 40% lower error than analog PET on the TOF images optimized for SNR.

### Illustrating images and supplemental figures

**Fig. 4:**
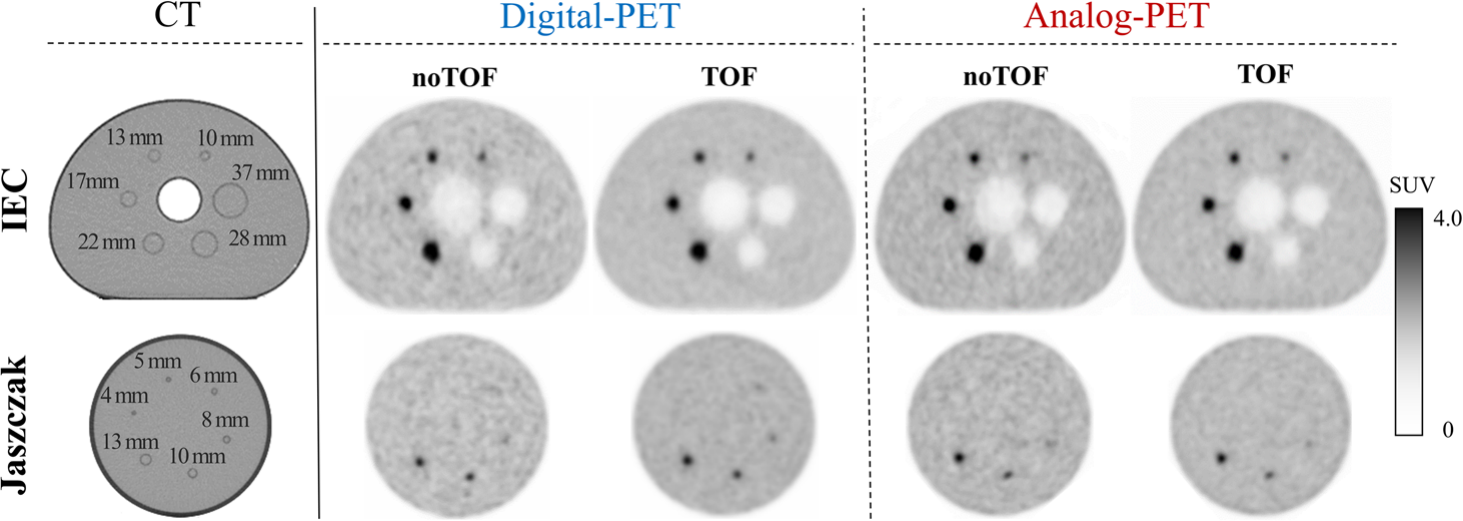
Cross-sectional slices passing through the spheres from the IEC and Jaszczak phantom (upper and lower panels, respectively) and recorded with CT, as well as with digital PET and analog PET and with reconstructions maximizing the SNR of the 10 mm diameter sphere with the TOF information (TOF) or without the latter (noTOF)

As evidenced in Fig. 4, the smallest hot spheres from the Jaszczak phantom, which were identifiable over the noise on the TOF images optimized for SNR, was the 6-mm diameter sphere for digital PET and the 8-mm diameter sphere for analog PET. Figures 2 and 4 also show that the visual detectability of, respectively, the adenopathies and small spheres was markedly enhanced on TOF than on noTOF images, especially for digital PET.

Supplemental Figures 5 to 7 depict additional analyses according to the number of iterations used for image reconstruction of CRC and SNR data from the IEC phantom (Supplemental Figure 5) and from human images (Supplemental Figure 6), with additional human PET images being displayed for OSEM iteration values ranging from 1 to 4 (Supplemental Figure 7).

Lastly, as evidenced in Supplemental Figure 8, no notable edge artifacts (also known as Gibbs artifacts) was documented on either the TOF and noTOF images from both cameras on the larger 37-mm hot sphere and for various representative levels of SNR (15, 20, 25).

## Discussion

The present study shows that image SNR from digital PET images, when maximized through reconstruction parameters, may be dramatically higher than that from analog PET, a superiority that is markedly underestimated by conventional NEMA-based analyses.

Marked improvements in image noise and contrast had already been documented several years ago in studies reporting the introduction of TOF PET imaging [3–5]. A similar finding was obtained herein with the comparison between digital and analog PET, but at a higher level of image quality. When using the TOF information, as well as when enhancing this information through better TOF resolutions, the convergence of the iterative process of image reconstruction is likely to be reached much faster, along with the use of less iterations, as observed in the present study. On the other hand, the use of less iterations is also associated with lower image noise. Hence, a faster contrast convergence associated with digital PET is likely to lead to a more advantageous trade-off between lesion contrast and image noise [5, 6]. Accordingly, the trade-off between contrast and SNR was able to be thoroughly investigated in phantom and human images through a wide-ranging number of OSEM iterations in the present study, and found to be consistently more advantageous for the TOF images from digital PET than for those from analog PET, with the difference being particularly pronounced for the smallest elements and the lowest numbers of iterations (Figs. 1, 2). In contrast, when the iteration number increased, the SNR/contrast trade-off provided by TOF images was found to converge with that observed with the noTOF images (see Figs. 1, 2); in other words, the use of a limited number of iterations is required to take full advantage of the TOF information.

A number of developments are currently being pursued for lowering the injected activities and/or PET recording times [29–31]. Recent studies have proposed appropriate regularization schemes to more effectively limit the increase in noise level [32, 33]. However, the present study strengthens the general consideration that the current objectives of decreasing administered activities and/or recording times could take advantage of the enhancements in TOF resolution and image quality provided by newly commercialized digital PET cameras. To the best of our knowledge, the present is the first study in which this enhancement is directly assessed through a head-to-head comparison with an analog PET camera equipped with modern and equivalent algorithms for image reconstruction.

The physical properties of the Ingenuity and Vereos cameras had already been described separately under NEMA-standard conditions [9, 10, 18]. Based on these previous NEMA-based analyses and on those from the present study, a significant advantage could be documented for image quality from the Vereos digital PET camera, but mainly for the CRC and SNR from small hot spheres (see Fig. 1 and supplemental Tables). Furthermore, in these NEMA-conditions, the relative difference in SNR between analog and digital PET was limited, ranging from − 4 to + 12% for the largest to the smallest IEC spheres.

Although the use of NEMA standards is mandatory for comparing the physical properties of PET cameras, it is likely that the NEMA-based reconstructions favor image contrast at the expense of SNR, especially for the Vereos camera (see Figs. 1, 3a). The NEMA-based reconstruction of the Ingenuity images involves an additional relaxation parameter of 0.5, applied in the image update step of the OSEM algorithm, which leads to small changes in the trade-off between noise and contrast although in a different manner according to sphere size (i.e., with an improvement for the 10-mm sphere but a worsening for the larger spheres, as evidenced in Fig. 1).

Nevertheless, these NEMA-based reconstructions are associated with rather low levels of SNR, as evidenced in Fig. 1a-c, and in these conditions, further decreases in SNR are likely susceptible to impair the detectability of small lesions. Hence, other reconstruction parameters favoring SNR would be more suitable when the injected activities and/or recording times are decreased, although this remains to be fully established through dedicated clinical trials. Notwithstanding the latter, the present study provides compelling evidence of a dramatic superiority of digital PET for such reconstructions favoring SNR.

In view of this latter finding, an optimization process, based on the maximization of SNR from small elements (i.e., spheres and adenopathies) [3, 4], was consequently applied on both cameras. Such maximization is indeed likely to define an optimal trade-off between contrast obtained in small lesions and noise level, and thus to reflect the visual ability to distinguish small lesions over noise [3, 4]. Although SNR is known to be strongly linked to the amount of recorded counts, we have observed that the number of iterations providing the maximal SNR remains unchanged for a very large range of recorded counts (results not shown). For the present PET images reconstructed with the TOF information and optimized for SNR, the advantage of digital over analog PET became substantial, with an almost 30% lower noise and, according to sphere size, with a 0 to 10% higher contrast as well as a 37% to 44% higher SNR. The number of iterations maximizing SNR was documented for both cameras at levels where the convergences for contrast were not achieved, (Fig. 1a-c). However, while contrast would likely be increased by using a higher number of iterations, this would be achieved at the expense of decreases in SNR of small elements.

This latter point is best illustrated in our human TOF images obtained with the low injected activity of 3 MBg kg^−1^ FDG and with a 90 s recording time per bed position. The small axillary adenopathies were well delineated with 1 and 2 iterations for digital and analog PET respectively, as shown in Fig. 2. By contrast, as evidenced in Supplemental Figure 7, a marked and seemingly deleterious increase in noise level could be observed when additional iterations were used to reconstruct the TOF images from both cameras. However, it should be recognized that these considerations are partly subjective, depending on the varying levels of noise accepted by physicians, and as already stated above, their potential consequences on medical diagnosis need to be precisely quantified.

Another key observation in the present study was that the maximal SNR level achieved on the 10-mm sphere with the digital PET images reconstructed without the TOF information was rather similar to that from analog PET. This provides conclusive evidence that the superiority of digital PET in reaching high maximal SNR levels for small elements is mostly attributable to the enhancement in TOF resolution when compared with analog PET.

The advantages provided by the better TOF resolution from digital PET allow enhancing not only the detectability of small lesions but also the accuracy by which larger lesions may be characterized and quantified [15, 16], particularly larger cold lesions. This last consideration is illustrated by our observation that both the SNR of the 37-mm cold sphere and the errors in the correction of attenuation and scatter were markedly enhanced on the optimized TOF images from digital PET, as compared with analog PET, whereas such enhancement was no longer documented on the corresponding noTOF images (Fig. 3).

Although the maximal levels of SNR remained relatively similar between the noTOF images from both cameras, it should be pointed out that these maximal ratios were reached with slightly higher values for noise and particularly for contrast of small spheres with digital PET. This higher noise is likely related to the lower count sensitivity of the Vereos digital PET (5200 vs. 7390 counts/s/MBq [10, 18]) than those from the Ingenuity analog camera, in keeping with a smaller axial field of view, shorter crystals and a smaller energy window (164 vs. 276 keV). In addition, the higher contrast documented for small spheres, together with a slightly higher SNR, may be at least partly attributed to an enhanced spatial resolution (4.2 vs. 4.8 mm full width at half maximum at the field of view center [10, 18]) and likely explained by the 1:1 dSiPM/crystal coupling and the smaller transversal field of view (76.4 vs. 90.3 cm) of the Vereos PET.

In contrast to that observed for the hot spheres, the CRC and the SNR documented on the noTOF images of the large cold sphere were lower with digital PET comparatively with analog PET. This could putatively be explained by the larger amount of noise on the noTOF images from digital PET and additionally, by the greater influence of this noise level on the SNR and CRC values from cold spheres as opposed to hot spheres.

In this study, a PSF algorithm was applied on all reconstructed images, with parameters currently recommended by the manufacturer for routine clinical exams. However, it may be pointed out that the differences detailed herein between TOF and noTOF images and between the two systems remained unchanged for images reconstructed without PSF (results not shown). In addition, it is well known that PSF algorithms may cause Gibbs artifacts, which appear as an overshoot and ringing signal within borders, in line with a sharp intensity transition in the image, leading to bias in image quantitation [34–36]. However, no Gibbs artifact was clearly documented on the 37-mm hot sphere (see Supplemental Figure 8), in accordance with that already reported in the literature for the same PSF parameters [37].

In addition, it should be kept in mind that optimal reconstruction parameters may also depend on the patient’s anthropometric parameters, as well as on tracer type and pharmacokinetics. For example, the introduction of TOF-PET imaging was previously shown to be particularly advantageous in obese patients [38] and such advantage should likely be even higher with the enhanced TOF information provided by digital PET.

However, the present results should invariably be confirmed by additional observations under various conditions. In particular, as stated above, dedicated clinical studies, performed on a large panel of patients, are required for providing a more extensive and more objective analysis.

In conclusion, this study shows that image quality is dramatically enhanced for digital PET, as compared with analog PET, for images optimized for the SNR from small lesions, the latter of which may prove particularly useful for PET exams performed with reduced injected activities and/or recording times. This superiority, which is much higher than that observed with NEMA standard-based reconstruction parameters, is mostly attributable to enhancements in TOF resolution and in inherent noise properties.

## Supplementary information

**Additional file 1:** **Supplemental Figure 5.** Results from the IEC phantom with the comparisons between digital-PET (solid lines) and analog-PET (dashed lines), for noTOF (left panels) and TOF images (right panels) and according of the number of OSEM iterations, of the Contrast Recovery Coefficients (CRC, upper panels) and Signal-to-Noise-Ratios (SNR, lower panels) of the hot spheres of 10, 22 and 37-mm diameters of the IEC phantom (red, purple and black lines, respectively). The number of iterations maximizing the SNR of the 10 mm sphere is indicated by vertical solid lines for digital-PET and dashed lines for analog-PET

**Additional file 2:** **Supplemental Figure 6.** Results from human PET images with the comparisons between noTOF (dashed lines) and TOF (solid lines) images and for both digital-PET (left panels) and analog-PET (right panels), of the contrast values and SNR determined for axillary adenopathies of 8 to 9 mm diameter, as a function of the number of OSEM iterations. The number of iterations maximizing the SNR of the axillary adenopathy is indicated by vertical solid lines for digital-PET and dashed lines for analog-PET

**Additional file 3:** **Supplemental Figure 7.** Maximal intensity projections of human PET images reconstructed with the TOF information for both digital-PET (upper panel) and analog-PET (lower panel) and with a number of OSEM iterations ranging from 1 to 4. Images corresponding to the maximal SNR the 10 mm sphere are surrounded by borders

**Additional file 4:** **Supplemental Figure 8.** Digital-PET (left panel) and analog-PET (right panel) images of the hot sphere of 37-mm diameter corresponding to three different levels of Signal-to-Noise-Ratio (SNR of 25, 20 and 15, respectively)

**Additional file 5:** **Supplemental Table 1.** Mean and standard deviation of SNR values (3 replicates) obtained for all spheres of the IEC phantoms visualized by both cameras (≥ 8-mm in diameter). Values are given for analog- and digital-PET images obtained with and without TOF (TOF and noTOF respectively) and with 1 to 10 OSEM iterations, as well as with NEMA-defined reconstruction parameters

**Additional file 6:** **Supplemental Table 2.** Mean and standard deviation of CRC values (3 replicates) obtained for all spheres of the IEC phantoms visualized by both cameras (≥ 8-mm in diameter). Values are given for analog- and digital-PET images obtained with and without TOF (TOF and noTOF respectively) and with 1 to 10 OSEM iterations, as well as with NEMA-defined reconstruction parameters

## Data Availability

The datasets used and/or analyzed during the current study are available from the corresponding author on reasonable request.
