# Supplementary figures and images for: Head-to-head comparison between digital and analog PET of human and phantom images when optimized for maximizing the signal-to-noise ratio from small lesions
Source: EJNMMI Phys. 2020 Feb 21;7:11. doi: 10.1186/s40658-020-0281-8 (PMC7035408; doi:10.1186/s40658-020-0281-8)

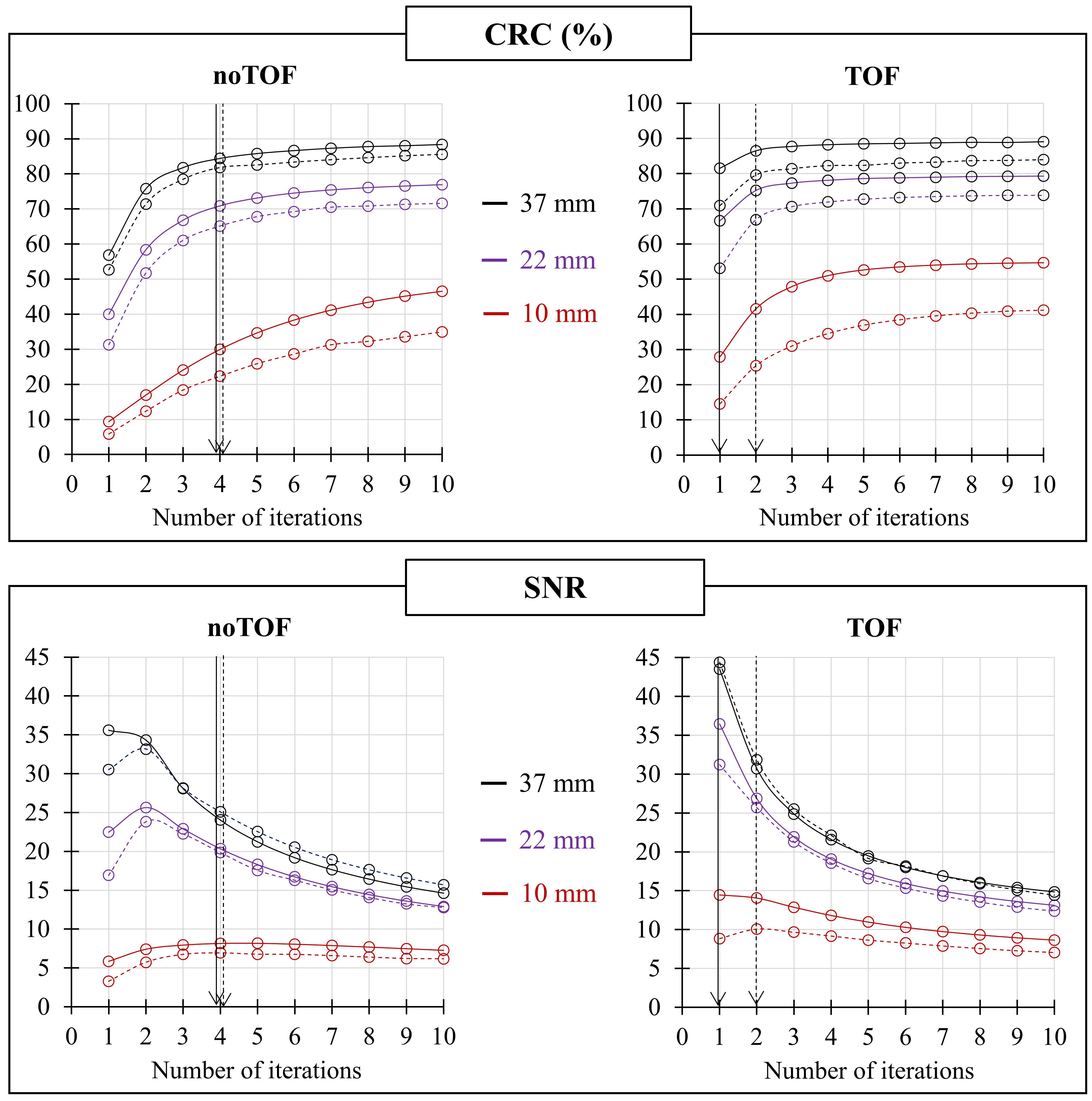

Supplement: Supplementary file 1 — Additional file 1: Supplemental Figure 5. Results from the IEC phantom with the comparisons between digital-PET (solid lines) and analog-PET (dashed lines), for noTOF (left panels) and TOF images (right panels) and according of the number of OSEM iterations, of the Contrast Recovery Coefficients (CRC, upper panels) and Signal-to-Noise-Ratios (SNR, lower panels) of the hot spheres of 10, 22 and 37-mm diameters of the IEC phantom (red, purple and black lines, respectively). The number of iterations maximizing the SNR of the 10 mm sphere is indicated by vertical solid lines for digital-PET and dashed lines for analog-PET [file 40658_2020_281_MOESM1_ESM.png]

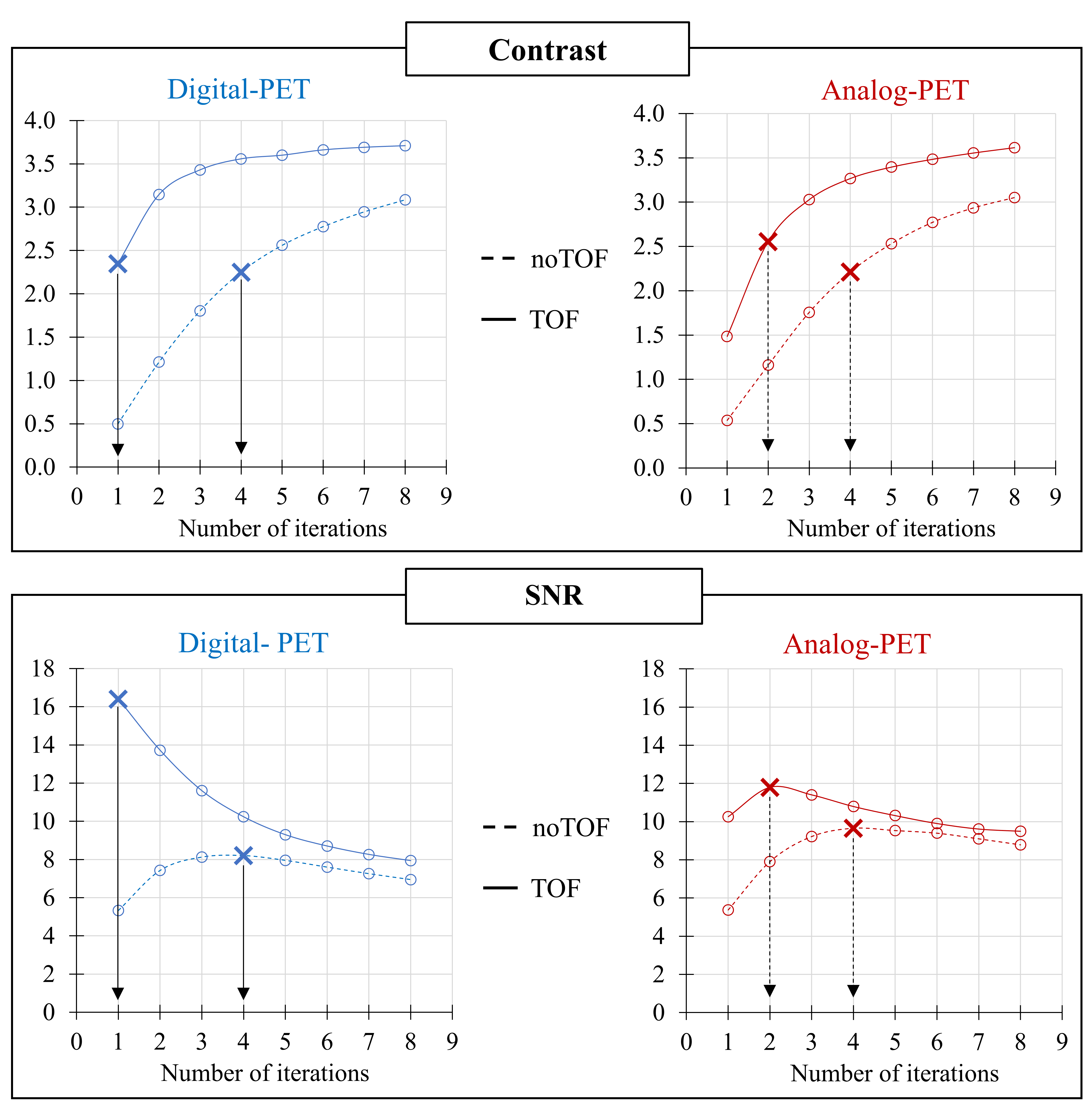

Supplement: Supplementary file 2 — Additional file 2: Supplemental Figure 6. Results from human PET images with the comparisons between noTOF (dashed lines) and TOF (solid lines) images and for both digital-PET (left panels) and analog-PET (right panels), of the contrast values and SNR determined for axillary adenopathies of 8 to 9 mm diameter, as a function of the number of OSEM iterations. The number of iterations maximizing the SNR of the axillary adenopathy is indicated by vertical solid lines for digital-PET and dashed lines for analog-PET [file 40658_2020_281_MOESM2_ESM.png]

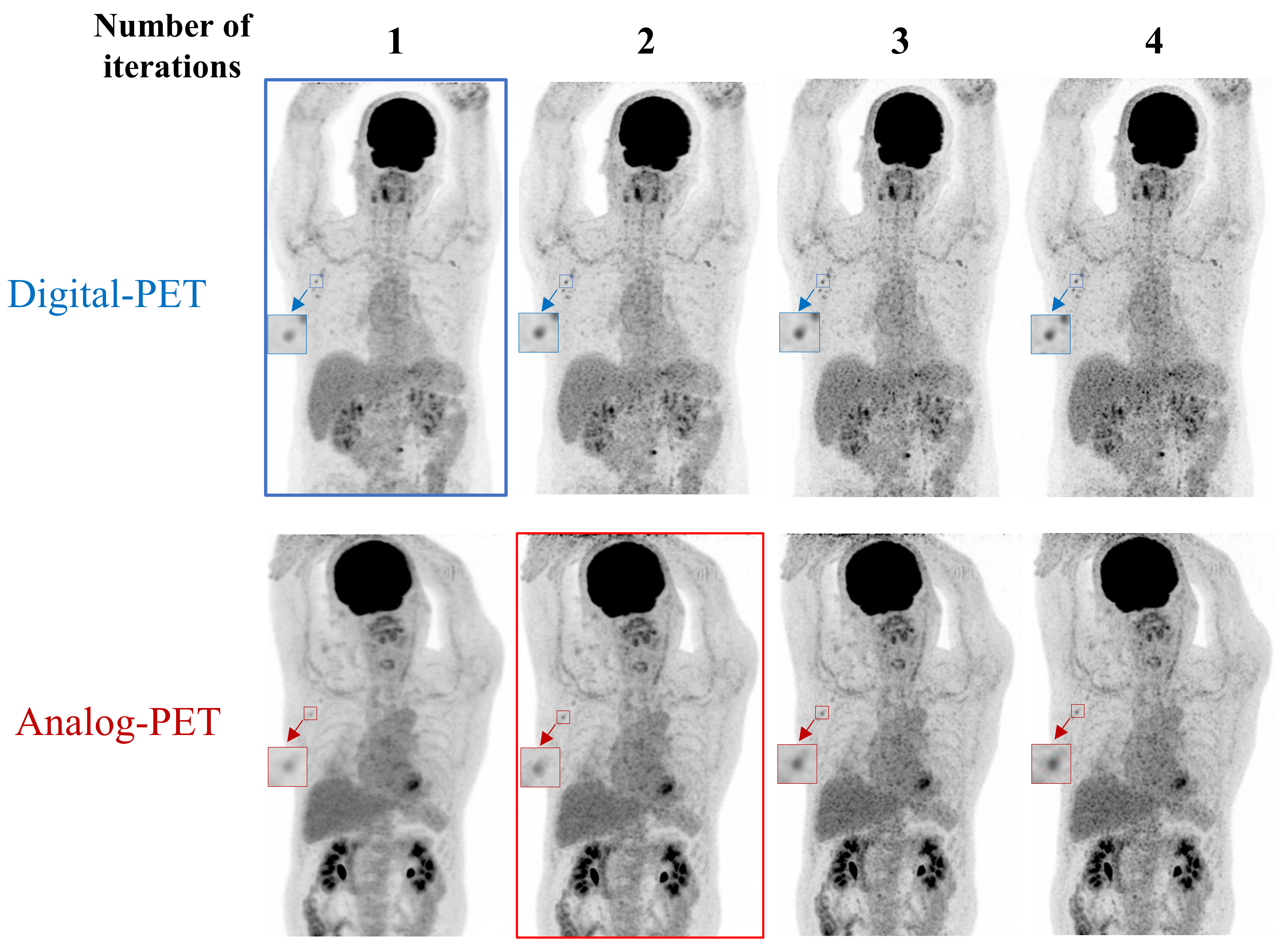

Supplement: Supplementary file 3 — Additional file 3: Supplemental Figure 7. Maximal intensity projections of human PET images reconstructed with the TOF information for both digital-PET (upper panel) and analog-PET (lower panel) and with a number of OSEM iterations ranging from 1 to 4. Images corresponding to the maximal SNR the 10 mm sphere are surrounded by borders [file 40658_2020_281_MOESM3_ESM.png]

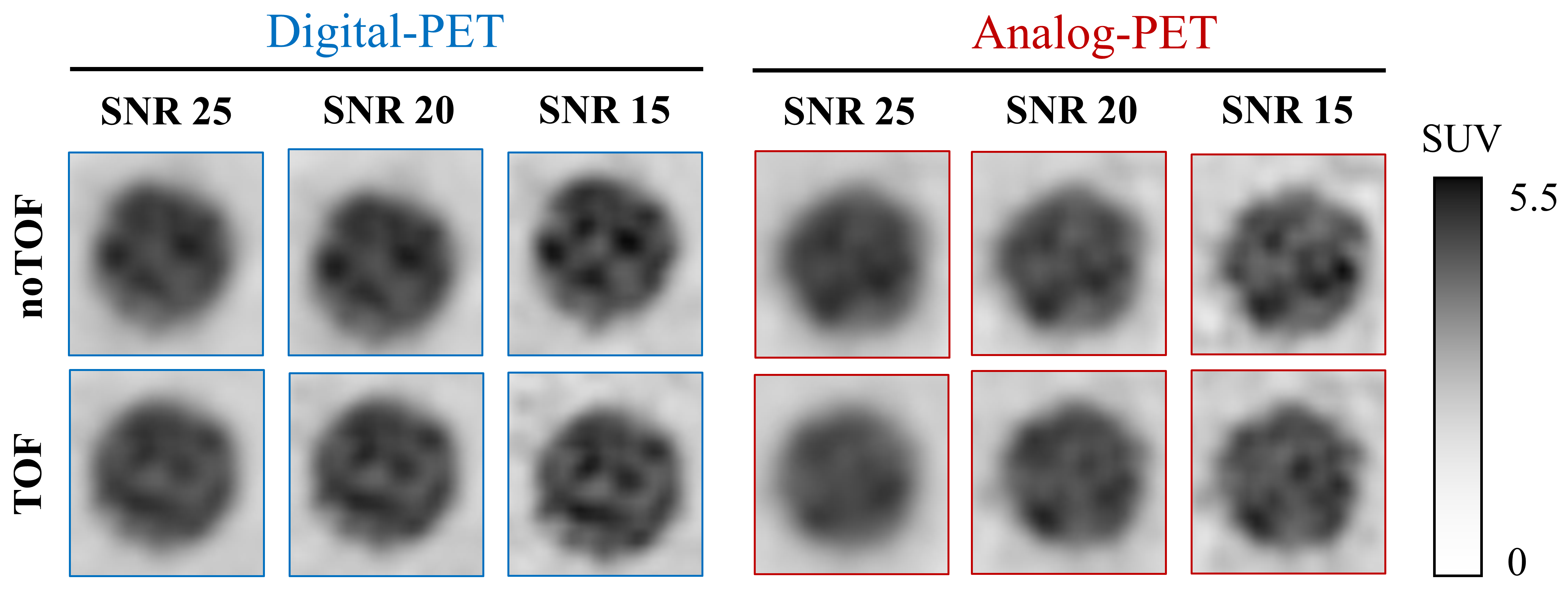

Supplement: Supplementary file 4 — Additional file 4: Supplemental Figure 8. Digital-PET (left panel) and analog-PET (right panel) images of the hot sphere of 37-mm diameter corresponding to three different levels of Signal-to-Noise-Ratio (SNR of 25, 20 and 15, respectively) [file 40658_2020_281_MOESM4_ESM.png]
