# Supplementary material for: Head-to-head comparison between digital and analog PET of human and phantom images when optimized for maximizing the signal-to-noise ratio from small lesions
Source: EJNMMI Phys. 2020 Feb 21;7:11. doi: 10.1186/s40658-020-0281-8 (PMC7035408; doi:10.1186/s40658-020-0281-8)
Supplement: Supplementary file 5 — Additional file 5: Supplemental Table 1. Mean and standard deviation of SNR values (3 replicates) obtained for all spheres of the IEC phantoms visualized by both cameras (≥ 8-mm in diameter). Values are given for analog- and digital-PET images obtained with and without TOF (TOF and noTOF respectively) and with 1 to 10 OSEM iterations, as well as with NEMA-defined reconstruction parameters [file 40658_2020_281_MOESM5_ESM.docx]

**Supplemental Table 1:** Mean and standard deviation of SNR values (3 replicates) obtained for all spheres of the IEC phantoms visualized by both cameras (≥ 8-mm in diameter). Values are given for analog- and digital-PET images obtained with and without TOF (TOF and noTOF respectively) and with 1 to 10 OSEM iterations, as well as with NEMA-defined reconstruction parameters.

| **SNR** | | | **Mean** | | | | | | | | | **SD** | | | | | | | | |
| --- | --- | --- | --- | --- | --- | --- | --- | --- | --- | --- | --- | --- | --- | --- | --- | --- | --- | --- | --- | --- |
|  |  |  | Hot sphere  diameters  (mm) | | | | | | | Cold sphere  diameters (mm) | | Hot sphere  diameters  (mm) | | | | | | | Cold sphere  diameters (mm) | |
|  | | **Number of Iterations** | **8** | **10** | **13** | **17** | **22** | **28** | **37** | **28** | **37** | **8** | **10** | **13** | **17** | **22** | **28** | **37** | **28** | **37** |
| **noTOF** | Digital | **1** | 4.3 | 5.9 | 8.9 | 16.6 | 22.5 | 27.4 | 35.6 | 8.1 | 7.9 | 0.5 | 1.8 | 1.0 | 2.2 | 3.4 | 0.9 | 3.0 | 1.3 | 1.0 |
|  |  | **2** | 4.4 | 7.4 | 12.8 | 19.6 | 25.6 | 29.1 | 34.3 | 7.6 | 7.9 | 0.5 | 0.8 | 1.1 | 1.6 | 1.5 | 1.1 | 2.3 | 0.7 | 0.5 |
|  |  | **3** | 4.3 | 8.0 | 13.5 | 18.7 | 22.9 | 25.5 | 28.1 | 6.4 | 6.9 | 0.5 | 0.8 | 0.9 | 1.2 | 0.9 | 0.7 | 1.6 | 0.4 | 0.3 |
|  |  | **4** | 4.2 | 8.2 | 13.3 | 17.3 | 20.4 | 22.5 | 24.0 | 5.7 | 6.1 | 0.4 | 0.7 | 0.8 | 1.0 | 0.7 | 0.5 | 1.2 | 0.3 | 0.2 |
|  |  | **5** | 4.0 | 8.2 | 12.8 | 16.0 | 18.3 | 20.1 | 21.2 | 5.1 | 5.6 | 0.4 | 0.6 | 0.7 | 0.8 | 0.6 | 0.3 | 0.9 | 0.3 | 0.2 |
|  |  | **6** | 3.9 | 8.1 | 12.1 | 14.8 | 16.7 | 18.3 | 19.2 | 4.7 | 5.2 | 0.4 | 0.5 | 0.6 | 0.7 | 0.5 | 0.2 | 0.7 | 0.2 | 0.2 |
|  |  | **7** | 3.7 | 7.9 | 11.5 | 13.8 | 15.5 | 17.0 | 17.7 | 4.4 | 4.8 | 0.4 | 0.4 | 0.6 | 0.6 | 0.5 | 0.1 | 0.6 | 0.2 | 0.2 |
|  |  | **8** | 3.6 | 7.7 | 10.9 | 12.9 | 14.5 | 15.8 | 16.4 | 4.2 | 4.6 | 0.4 | 0.4 | 0.5 | 0.6 | 0.4 | 0.0 | 0.5 | 0.2 | 0.1 |
|  |  | **9** | 3.5 | 7.5 | 10.4 | 12.2 | 13.6 | 14.9 | 15.4 | 4.0 | 4.3 | 0.4 | 0.3 | 0.5 | 0.5 | 0.4 | 0.0 | 0.4 | 0.2 | 0.1 |
|  |  | **10** | 3.4 | 7.3 | 9.9 | 11.6 | 12.9 | 14.1 | 14.6 | 3.8 | 4.1 | 0.4 | 0.3 | 0.5 | 0.5 | 0.3 | 0.1 | 0.4 | 0.2 | 0.1 |
|  | Analog | **1** | 4.1 | 3.3 | 5.9 | 12.5 | 16.9 | 18.6 | 30.5 | 7.5 | 8.1 | 0.3 | 2.6 | 4.1 | 4.1 | 5.9 | 0.8 | 2.1 | 0.5 | 0.1 |
|  |  | **2** | 4.2 | 5.7 | 11.1 | 17.9 | 23.8 | 27.0 | 33.2 | 8.2 | 9.0 | 0.3 | 2.4 | 2.1 | 1.8 | 1.9 | 1.9 | 1.6 | 0.4 | 0.7 |
|  |  | **3** | 4.2 | 6.8 | 12.3 | 17.9 | 22.3 | 25.4 | 28.2 | 7.2 | 8.0 | 0.3 | 2.1 | 1.0 | 1.3 | 1.8 | 1.8 | 2.5 | 0.3 | 0.5 |
|  |  | **4** | 4.1 | 6.9 | 12.2 | 16.8 | 19.9 | 22.7 | 25.1 | 6.4 | 7.1 | 0.3 | 1.9 | 0.5 | 1.3 | 1.7 | 1.6 | 2.2 | 0.3 | 0.5 |
|  |  | **5** | 4.0 | 6.8 | 11.6 | 15.3 | 17.6 | 20.4 | 22.5 | 5.5 | 6.2 | 0.3 | 1.7 | 0.5 | 1.5 | 2.1 | 1.4 | 2.0 | 0.6 | 0.8 |
|  |  | **6** | 3.9 | 6.8 | 11.2 | 14.5 | 16.3 | 18.6 | 20.5 | 5.3 | 6.0 | 0.2 | 1.6 | 0.4 | 1.2 | 1.4 | 1.3 | 1.8 | 0.3 | 0.4 |
|  |  | **7** | 3.8 | 6.6 | 10.6 | 13.5 | 15.1 | 17.1 | 18.9 | 5.0 | 5.6 | 0.2 | 1.6 | 0.4 | 1.1 | 1.3 | 1.2 | 1.7 | 0.2 | 0.4 |
|  |  | **8** | 3.8 | 6.4 | 10.0 | 12.7 | 14.1 | 15.9 | 17.6 | 4.7 | 5.3 | 0.2 | 1.5 | 0.4 | 1.0 | 1.2 | 1.1 | 1.6 | 0.2 | 0.4 |
|  |  | **9** | 3.7 | 6.2 | 9.6 | 12.0 | 13.3 | 15.0 | 16.6 | 4.5 | 5.0 | 0.2 | 1.4 | 0.4 | 1.0 | 1.1 | 1.0 | 1.5 | 0.2 | 0.4 |
|  |  | **10** | 3.7 | 6.2 | 9.2 | 11.7 | 12.8 | 14.2 | 15.7 | 4.4 | 4.9 | 0.2 | 1.4 | 0.4 | 0.9 | 0.8 | 1.0 | 1.4 | 0.0 | 0.1 |
| **TOF** | Digital | **1** | 7.1 | 14.5 | 24.0 | 30.6 | 36.5 | 40.1 | 43.5 | 10.6 | 12.0 | 0.8 | 1.5 | 1.0 | 1.2 | 0.5 | 0.0 | 0.9 | 0.1 | 0.2 |
|  |  | **2** | 7.2 | 14.1 | 20.3 | 24.0 | 26.9 | 29.0 | 30.7 | 8.1 | 8.8 | 0.8 | 1.4 | 0.8 | 1.1 | 0.8 | 0.3 | 0.4 | 0.2 | 0.2 |
|  |  | **3** | 6.9 | 12.9 | 17.3 | 19.9 | 21.9 | 23.6 | 24.9 | 6.8 | 7.3 | 0.7 | 1.2 | 0.7 | 0.9 | 0.7 | 0.3 | 0.2 | 0.2 | 0.2 |
|  |  | **4** | 6.6 | 11.8 | 15.3 | 17.4 | 19.1 | 20.5 | 21.6 | 6.1 | 6.5 | 0.7 | 1.1 | 0.6 | 0.8 | 0.7 | 0.3 | 0.1 | 0.2 | 0.2 |
|  |  | **5** | 6.2 | 11.0 | 14.0 | 15.7 | 17.2 | 18.5 | 19.5 | 5.6 | 5.9 | 0.7 | 1.0 | 0.6 | 0.8 | 0.6 | 0.3 | 0.1 | 0.2 | 0.2 |
|  |  | **6** | 5.9 | 10.3 | 13.0 | 14.5 | 15.9 | 17.1 | 18.0 | 5.2 | 5.5 | 0.6 | 0.9 | 0.6 | 0.7 | 0.6 | 0.3 | 0.0 | 0.2 | 0.2 |
|  |  | **7** | 5.7 | 9.7 | 12.2 | 13.7 | 15.0 | 16.1 | 16.9 | 5.0 | 5.2 | 0.6 | 0.8 | 0.5 | 0.7 | 0.5 | 0.3 | 0.0 | 0.1 | 0.2 |
|  |  | **8** | 5.5 | 9.3 | 11.6 | 13.0 | 14.2 | 15.3 | 16.1 | 4.7 | 4.9 | 0.6 | 0.8 | 0.5 | 0.7 | 0.5 | 0.3 | 0.0 | 0.1 | 0.2 |
|  |  | **9** | 5.3 | 8.9 | 11.1 | 12.5 | 13.6 | 14.7 | 15.4 | 4.6 | 4.7 | 0.6 | 0.7 | 0.5 | 0.6 | 0.5 | 0.3 | 0.0 | 0.1 | 0.2 |
|  |  | **10** | 5.1 | 8.6 | 10.8 | 12.0 | 13.1 | 14.1 | 14.8 | 4.4 | 4.6 | 0.5 | 0.7 | 0.5 | 0.6 | 0.5 | 0.3 | 0.0 | 0.1 | 0.2 |
|  |  | **NEMA** |  | 10.8 | 12.8 | 15.4 | 16.3 | 18.0 | 18.9 | 5.5 | 5.9 |  | 1.3 | 0.8 | 0.7 | 0.8 | 0.3 | 0.2 | 0.2 | 0.2 |
|  | Analog | **1** | 4.3 | 8.8 | 16.7 | 23.9 | 31.2 | 37.5 | 44.4 | 11.0 | 12.3 | 0.3 | 1.6 | 1.6 | 1.8 | 2.0 | 1.5 | 2.6 | 0.0 | 0.3 |
|  |  | **2** | 4.8 | 10.1 | 16.9 | 22.1 | 25.7 | 29.1 | 31.9 | 7.8 | 8.6 | 0.3 | 1.6 | 1.5 | 1.3 | 1.4 | 1.2 | 1.8 | 0.9 | 0.8 |
|  |  | **3** | 4.9 | 9.7 | 15.2 | 19.0 | 21.3 | 23.6 | 25.5 | 6.8 | 7.4 | 0.3 | 1.6 | 1.4 | 1.1 | 1.1 | 0.9 | 1.5 | 0.5 | 0.4 |
|  |  | **4** | 5.0 | 9.2 | 13.7 | 16.8 | 18.6 | 20.5 | 22.1 | 6.2 | 6.7 | 0.3 | 1.5 | 1.2 | 0.9 | 1.0 | 0.8 | 1.3 | 0.3 | 0.2 |
|  |  | **5** | 4.9 | 8.6 | 12.5 | 15.1 | 16.6 | 18.0 | 19.1 | 5.7 | 6.1 | 0.3 | 1.4 | 1.1 | 0.8 | 1.0 | 0.7 | 1.1 | 0.2 | 0.0 |
|  |  | **6** | 4.9 | 8.3 | 11.7 | 14.0 | 15.4 | 16.8 | 18.2 | 5.3 | 5.7 | 0.3 | 1.3 | 1.0 | 0.8 | 0.9 | 0.7 | 1.0 | 0.1 | 0.0 |
|  |  | **7** | 4.8 | 7.9 | 11.0 | 13.1 | 14.3 | 15.6 | 16.9 | 5.0 | 5.4 | 0.3 | 1.2 | 1.0 | 0.7 | 0.8 | 0.6 | 1.0 | 0.1 | 0.0 |
|  |  | **8** | 4.8 | 7.6 | 10.4 | 12.4 | 13.5 | 14.7 | 15.9 | 4.8 | 5.1 | 0.3 | 1.2 | 0.9 | 0.7 | 0.8 | 0.6 | 0.9 | 0.1 | 0.1 |
|  |  | **9** | 4.7 | 7.3 | 9.9 | 11.8 | 12.9 | 14.0 | 15.1 | 4.6 | 4.9 | 0.3 | 1.1 | 0.9 | 0.6 | 0.8 | 0.6 | 0.9 | 0.0 | 0.1 |
|  |  | **10** | 4.7 | 7.0 | 9.6 | 11.3 | 12.4 | 13.3 | 14.4 | 4.5 | 4.8 | 0.3 | 1.1 | 0.8 | 0.6 | 0.7 | 0.5 | 0.8 | 0.0 | 0.1 |
|  |  | **NEMA** |  | 9.6 | 13.2 | 15.7 | 17 | 18.4 | 19.8 | 6.1 | 6.5 |  | 0.7 | 1.3 | 0.9 | 0.6 | 0.3 | 0.3 | 0.1 | 0.3 |
