# Supplementary material for: Head-to-head comparison between digital and analog PET of human and phantom images when optimized for maximizing the signal-to-noise ratio from small lesions
Source: EJNMMI Phys. 2020 Feb 21;7:11. doi: 10.1186/s40658-020-0281-8 (PMC7035408; doi:10.1186/s40658-020-0281-8)
Supplement: Supplementary file 6 — Additional file 6: Supplemental Table 2. Mean and standard deviation of CRC values (3 replicates) obtained for all spheres of the IEC phantoms visualized by both cameras (≥ 8-mm in diameter). Values are given for analog- and digital-PET images obtained with and without TOF (TOF and noTOF respectively) and with 1 to 10 OSEM iterations, as well as with NEMA-defined reconstruction parameters [file 40658_2020_281_MOESM6_ESM.docx]

**Supplemental Table 2:** Mean and standard deviation of CRC values (3 replicates) obtained for all spheres of the IEC phantoms visualized by both cameras (≥ 8-mm in diameter). Values are given for analog- and digital-PET images obtained with and without TOF (TOF and noTOF respectively) and with 1 to 10 OSEM iterations, as well as with NEMA-defined reconstruction parameters.

| **CRC (%)** | | | **Mean** | | | | | | | | | **SD** | | | | | | | | |
| --- | --- | --- | --- | --- | --- | --- | --- | --- | --- | --- | --- | --- | --- | --- | --- | --- | --- | --- | --- | --- |
|  |  |  | Hot sphere  diameters  (mm) | | | | | | | Cold sphere  diameters (mm) | | Hot sphere  diameters  (mm) | | | | | | | Cold sphere  diameters (mm) | |
|  | | **Number of Iterations** | **8** | **10** | **13** | **17** | **22** | **28** | **37** | **28** | **37** | **8** | **10** | **13** | **17** | **22** | **28** | **37** | **28** | **37** |
| **noTOF** | Digital | **1** | 6.5 | 9.4 | 14.1 | 26.1 | 40.0 | 40.9 | 56.8 | 40.1 | 39.0 | 0.9 | 2.8 | 1.0 | 0.9 | 13.2 | 0.7 | 0.6 | 1.7 | 0.2 |
|  |  | **2** | 11.0 | 17.0 | 28.5 | 43.4 | 58.4 | 64.4 | 75.8 | 50.4 | 52.8 | 1.5 | 2.9 | 1.7 | 1.7 | 8.1 | 1.1 | 0.8 | 1.3 | 0.6 |
|  |  | **3** | 14.6 | 24.1 | 40.0 | 54.7 | 66.8 | 74.3 | 81.8 | 56.3 | 60.5 | 1.9 | 4.1 | 2.0 | 2.1 | 4.9 | 1.4 | 0.7 | 1.0 | 0.9 |
|  |  | **4** | 17.4 | 30.0 | 47.8 | 61.5 | 70.9 | 78.9 | 84.4 | 60.5 | 65.6 | 2.3 | 5.2 | 2.2 | 2.2 | 3.3 | 1.4 | 0.8 | 0.8 | 0.9 |
|  |  | **5** | 19.6 | 34.7 | 52.8 | 65.4 | 73.1 | 81.4 | 85.8 | 63.4 | 69.0 | 2.6 | 6.1 | 2.2 | 2.2 | 2.4 | 1.4 | 0.8 | 0.8 | 0.8 |
|  |  | **6** | 21.4 | 38.3 | 56.1 | 67.8 | 74.6 | 82.9 | 86.7 | 65.7 | 71.6 | 2.8 | 6.8 | 2.3 | 2.1 | 1.9 | 1.5 | 0.8 | 0.9 | 1.1 |
|  |  | **7** | 22.8 | 41.2 | 58.3 | 69.2 | 75.5 | 83.9 | 87.3 | 67.7 | 73.7 | 3.0 | 7.4 | 2.3 | 2.1 | 1.6 | 1.6 | 0.8 | 1.1 | 0.9 |
|  |  | **8** | 23.9 | 43.4 | 59.8 | 70.2 | 76.1 | 84.7 | 87.8 | 69.3 | 75.2 | 3.2 | 7.9 | 2.3 | 2.1 | 1.4 | 1.6 | 0.7 | 1.1 | 1.0 |
|  |  | **9** | 24.8 | 45.2 | 60.9 | 70.8 | 76.6 | 85.1 | 88.1 | 70.5 | 76.5 | 3.3 | 8.2 | 2.3 | 2.1 | 1.4 | 1.6 | 0.7 | 1.1 | 1.1 |
|  |  | **10** | 25.6 | 46.6 | 61.8 | 71.3 | 76.9 | 85.6 | 88.4 | 71.8 | 77.7 | 3.4 | 8.5 | 2.3 | 2.1 | 1.3 | 1.8 | 0.6 | 1.1 | 1.0 |
|  | Analog | **1** | 5.0 | 5.9 | 10.8 | 23.7 | 31.3 | 36.2 | 52.6 | 43.4 | 44.2 | 0.5 | 4.4 | 6.8 | 6.7 | 11.3 | 0.7 | 0.0 | 1.5 | 0.3 |
|  |  | **2** | 8.6 | 12.4 | 23.5 | 39.0 | 51.7 | 59.5 | 71.4 | 53.2 | 58.0 | 0.9 | 6.9 | 7.6 | 7.3 | 9.0 | 1.1 | 3.3 | 1.5 | 0.4 |
|  |  | **3** | 11.3 | 18.4 | 32.9 | 49.3 | 61.0 | 69.9 | 78.5 | 58.4 | 65.1 | 1.2 | 7.6 | 6.2 | 5.1 | 7.3 | 1.3 | 0.0 | 1.5 | 0.4 |
|  |  | **4** | 13.5 | 22.3 | 39.3 | 55.6 | 65.1 | 75.5 | 81.8 | 62.4 | 69.9 | 1.4 | 8.2 | 4.7 | 3.2 | 6.3 | 1.3 | 0.1 | 1.6 | 0.6 |
|  |  | **5** | 15.3 | 25.9 | 43.6 | 59.4 | 67.8 | 77.5 | 82.6 | 65.1 | 73.2 | 1.6 | 8.2 | 3.6 | 2.1 | 6.1 | 1.4 | 0.5 | 1.9 | 0.9 |
|  |  | **6** | 16.9 | 28.7 | 46.4 | 61.8 | 69.3 | 79.2 | 83.4 | 67.5 | 75.8 | 1.7 | 8.3 | 3.1 | 1.4 | 5.9 | 1.5 | 0.1 | 1.7 | 0.6 |
|  |  | **7** | 18.2 | 31.3 | 48.3 | 63.3 | 70.5 | 79.7 | 84.1 | 69.2 | 77.7 | 1.8 | 8.1 | 2.8 | 1.0 | 6.0 | 1.6 | 0.0 | 1.9 | 0.8 |
|  |  | **8** | 19.4 | 32.3 | 49.6 | 64.4 | 70.8 | 81.0 | 84.6 | 70.8 | 79.2 | 2.0 | 8.5 | 2.6 | 0.8 | 5.7 | 1.5 | 0.1 | 1.8 | 0.7 |
|  |  | **9** | 20.4 | 33.6 | 50.5 | 65.2 | 71.3 | 81.6 | 85.2 | 72.2 | 80.6 | 2.1 | 8.6 | 2.5 | 0.8 | 5.7 | 1.5 | 0.1 | 2.1 | 1.0 |
|  |  | **10** | 21.3 | 35.0 | 51.1 | 65.8 | 71.6 | 81.9 | 85.6 | 73.0 | 81.4 | 2.2 | 8.6 | 2.6 | 0.7 | 5.7 | 1.5 | 0.1 | 1.8 | 0.6 |
| **TOF** | Digital | **1** | 14.9 | 27.9 | 43.8 | 57.1 | 66.6 | 75.4 | 81.5 | 58.5 | 67.8 | 2.0 | 3.0 | 1.4 | 0.5 | 0.4 | 0.9 | 0.7 | 1.1 | 0.3 |
|  |  | **2** | 23.2 | 41.5 | 56.8 | 68.6 | 75.2 | 81.9 | 86.5 | 68.6 | 76.7 | 3.1 | 4.5 | 1.2 | 0.6 | 0.5 | 1.4 | 0.4 | 1.1 | 0.4 |
|  |  | **3** | 27.9 | 47.9 | 60.9 | 71.5 | 77.3 | 83.4 | 87.7 | 73.5 | 80.6 | 3.7 | 5.0 | 1.2 | 0.8 | 0.5 | 1.3 | 0.5 | 1.2 | 0.4 |
|  |  | **4** | 30.6 | 51.0 | 62.6 | 72.5 | 78.1 | 84.0 | 88.2 | 76.2 | 82.6 | 4.1 | 5.2 | 1.1 | 0.9 | 0.6 | 1.2 | 0.6 | 1.3 | 0.5 |
|  |  | **5** | 32.2 | 52.6 | 63.4 | 73.0 | 78.6 | 84.3 | 88.5 | 78.0 | 84.0 | 4.3 | 5.3 | 1.2 | 1.0 | 0.7 | 1.2 | 0.6 | 1.3 | 0.4 |
|  |  | **6** | 32.9 | 53.5 | 63.8 | 73.3 | 78.8 | 84.5 | 88.6 | 79.1 | 84.7 | 4.4 | 5.4 | 1.2 | 0.9 | 0.7 | 1.2 | 0.6 | 1.4 | 0.5 |
|  |  | **7** | 33.7 | 54.0 | 64.1 | 73.5 | 79.0 | 84.8 | 88.8 | 79.9 | 85.4 | 4.5 | 5.5 | 1.2 | 1.0 | 0.8 | 1.2 | 0.6 | 1.3 | 0.3 |
|  |  | **8** | 34.0 | 54.3 | 64.3 | 73.6 | 79.1 | 85.0 | 88.9 | 80.5 | 85.8 | 4.5 | 5.6 | 1.2 | 1.0 | 0.8 | 1.0 | 0.8 | 1.4 | 0.5 |
|  |  | **9** | 34.1 | 54.5 | 64.4 | 73.7 | 79.2 | 85.0 | 88.9 | 80.9 | 86.1 | 4.5 | 5.6 | 1.2 | 1.0 | 0.9 | 1.2 | 0.6 | 1.4 | 0.5 |
|  |  | **10** | 34.4 | 54.7 | 64.5 | 73.8 | 79.3 | 85.2 | 89.1 | 81.1 | 86.2 | 4.6 | 5.6 | 1.2 | 1.0 | 0.8 | 1.2 | 0.7 | 1.4 | 0.4 |
|  |  | **NEMA** | -- | 51.3 | 60.9 | 72.9 | 77.2 | 83.6 | 88.0 | 78.5 | 84.0 | -- | 5.2 | 5.4 | 2.2 | 2.8 | 2.2 | 0.6 | 2.1 | 1.0 |
|  | Analog | **1** | 8.1 | 14.6 | 27.5 | 40.1 | 53.1 | 61.4 | 70.9 | 53.2 | 59.6 | 0.8 | 2.8 | 0.7 | 1.1 | 2.8 | 1.2 | 0.2 | 1.7 | 0.3 |
|  |  | **2** | 13.5 | 25.4 | 42.4 | 56.8 | 66.9 | 74.4 | 79.6 | 62.8 | 69.4 | 1.4 | 4.5 | 1.4 | 1.4 | 3.7 | 1.5 | 0.3 | 1.9 | 0.4 |
|  |  | **3** | 17.4 | 31.0 | 48.6 | 62.2 | 70.6 | 77.4 | 81.4 | 68.1 | 74.5 | 1.8 | 5.5 | 1.6 | 1.4 | 3.9 | 1.6 | 0.4 | 2.0 | 0.3 |
|  |  | **4** | 20.3 | 34.5 | 51.8 | 64.3 | 72.0 | 79.1 | 82.3 | 71.1 | 77.3 | 2.1 | 6.1 | 9.6 | 1.5 | 3.9 | 1.7 | 0.4 | 2.3 | 0.5 |
|  |  | **5** | 22.6 | 36.9 | 53.2 | 65.3 | 72.7 | 79.0 | 82.4 | 73.6 | 79.5 | 2.3 | 6.8 | 1.9 | 1.5 | 3.8 | 1.7 | 0.3 | 2.7 | 0.8 |
|  |  | **6** | 24.4 | 38.4 | 54.1 | 66.0 | 73.2 | 80.3 | 83.0 | 75.0 | 80.7 | 2.5 | 6.8 | 1.8 | 1.5 | 4.0 | 1.7 | 0.6 | 2.4 | 0.6 |
|  |  | **7** | 25.8 | 39.5 | 54.7 | 66.4 | 73.5 | 80.4 | 83.2 | 76.1 | 81.6 | 2.6 | 7.0 | 1.8 | 1.5 | 4.0 | 1.7 | 0.6 | 2.5 | 0.6 |
|  |  | **8** | 27.0 | 40.3 | 55.1 | 66.7 | 73.7 | 80.9 | 83.7 | 77.0 | 82.3 | 2.7 | 7.2 | 1.8 | 1.5 | 4.0 | 1.7 | 0.6 | 2.6 | 0.6 |
|  |  | **9** | 27.9 | 40.9 | 55.4 | 66.9 | 73.8 | 81.0 | 83.7 | 77.7 | 82.9 | 2.8 | 7.3 | 1.8 | 1.5 | 4.0 | 1.7 | 0.6 | 2.6 | 0.7 |
|  |  | **10** | 28.7 | 41.2 | 55.5 | 66.9 | 73.8 | 81.2 | 83.9 | 77.9 | 83.1 | 2.9 | 7.2 | 1.8 | 1.5 | 4.0 | 1.7 | 0.6 | 2.6 | 0.7 |
|  |  | **NEMA** | -- | 37.8 | 53.4 | 63.3 | 68.5 | 73.2 | 78.6 | 72.7 | 78.8 | -- | 3.3 | 3.8 | 3.6 | 2.7 | 0.2 | 0.5 | 3.5 | 1.6 |
